# "The Hospital" Nursing Mirror

**Published:** 1901-04-13

**Authors:** 


"The Hospital" Nursing Mirror.

Being the Nursing Section of "The Hospital."

[Contributions for this Section of "The Hospital" should be addressed to the Editor, "The Hospital" Nursing Mirror, 28 & 29 Southampton Street Strand, London, W.C.]

### Notes on News from the Nursing World.

### HONOURS FOR ARMY NURSES.

The nurses who have saved lives for England in South Africa amd alleviated suffering are to receive the recognition which we suggested some months ago. Following the official announcement that the King has been graciously pleased to confirm the order given by her late Majesty Queen Victoria that a medal be struck to commemorate the military operations in South Africa, it is stated that the medal "in silver" will be granted not only to all officers and men of the British, Indian and Colonial forces, but also to "all nurses and nursing sisters who actually served in South Africa between October 11th, 1899, and a date to be hereafter fixed." Of course, the claims of each nurse will have to be approved by the Commander-in-chief, in whose judgment there will be absolute confidence; and we congratulate both the authorities and the army nurses on the manner in which the claims of the latter to honour are to be met. The award of special distinctions is not precluded, but it is right that all women whose services have been turned to account in the war should be placed on the same level as men.

### PRESENTATION OF THE RED CROSS MEDAL BY LORD KITCHENER.

Since Lord Kitchener has been commander-in-chief in South Africa he has not had many pleasant duties to perform. But a visit which he paid to Johannesburg on Thursday last week, doubtless afforded him unaffected satisfaction. It was undertaken for the purpose of presenting to that distinguished nurse, Madame Ferrières, the head of the French ambulance, the Red Cross medal, which has so very fitly been conferred on her for the useful assistance rendered by her to wounded British soldiers at the time of the occupation of the town. Lord Kitchener, in graceful language, performed his welcome task in No. 6 Field Hospital, and Madame Ferrières did not disguise the gratification with which she accepted the honour bestowed upon her.

### THE SUPPLY OF ARMY NURSES.

The Secretary of State for War informs us that the following members of the Army Nursing Service Reserve have left for South Africa in the hospital ship *Avoca*:—Misses E. F. Beedie, A. D. Cameron, A. M. Ferguson, S. A. Fisher, M. Hilson, M. E. Howell, A. M. Lashwood, M. B. Nuttall, D. Pryde, and H. Raggett. Miss Ferguson was trained at Longmore Hospital for Incurables, Edinburgh, and York County Hospital. She has since been attached to the Ancoats District Nurses' Home, district nurse at Settle, ward nurse and sister at St. George's Hospital, and from November 1896 she has been engaged in private nursing. Miss Helen Raggett was trained at St. Saviour's Infirmary, Dulwich, and for nearly two years has held the post of ward sister at Isleworth Infirmary, where she recently received instruction in massage, and last November passed the examination of Incorporated Society of Trained Masseuses. A further detachment of sisters of the Army Nursing Service Reserve, whose names are subjoined, will embark for South Africa about the 13th inst.:—Misses M. S. Barwell, J. Cameron, A. B. Cameron, C. Chapman, M. J. Cleghorn, J. Galloway, L. D. Hills, M. McLeish, L. D. Minton, and J. E. Mount. Some of these nurses have already seen service in South Africa, notably Miss M. S. Barwell, who has lately been on duty at the Military Hospital, Portobello, Dublin. It is not two months since one of these nurses sent in her application to join the Army Nursing Service Reserve. Does this mean that the supply is falling off? It seems to us that more should be enrolled as soon as possible in order that they may be ready in case of need.

### LORD RAGLAN'S DELUSION.

Mr. Sydney Holland, chairman of the London Hospital, writes to the *Times* to point out that the deputation from the "Matrons' Council," who waited upon the Under-Secretary for War last week to express their views on army nursing, "do not represent in any sense the nursing profession, or the matrons of the London or provincial hospitals." Mr. Holland continues: "They are a self-constituted body, not elected by the matrons of any of the principal hospitals, and with the exception of St. Bartholomew's, there is not, on the so-called 'Matrons' Council,' a single representative of a training school for nurses, even of second importance," and while he says that he has no objection to anyone trying to improve the army or any other nursing, he points out that the name taken "by these few ladies" gives "a fictitious importance to their views and probably led to their being received by the War Office at all." We we are not inclined to blame the "few ladies" for speaking in the name of the "Matrons' Council" which was the body they represented. The person to blame is the Under-Secretary for War, who should have taken the trouble to ascertain whether such a deputation had any claim to speak for the nursing profession before he consented to receive them. Of course Lord Raglan, as he pleaded a few days ago in the House of Lords, is new to the obligations of office, but if he had made a few inquiries, he would have saved himself from being the victim of the delusion that he was receiving an important representative body. The incident is only worth notice because it is as well that Mr. Holland's statement should be driven home in the department with which Lord Raglan is associated.

### AN APPEAL BY A NURSING SISTER IN SOUTH AFRICA.

An appeal is made, at the instance of Sister Borlase, by Miss S. Buchanan-Riddell, 9 Sloane Gardens, S.W., on behalf of the officers' hospital which has been opened by the Government at Johannesburg. The hospital is in charge of Miss Borlase, who, it will be remembered, was one of the two nurses who was in a train attacked by Boers, whose exciting adventures were related in our issue of last week. Writing from No. 1 General Hospital, Johannesburg, she says:—"It will be such a great help if I can get some nice things for the home. I should so like it to be for the time a real home, and with Government things one cannot do much. I should like nice soft bed linen and white blankets, pretty china—in fact, everything which kind hearts at home can provide by sending funds for comforts for the sick and wounded officers. There is a good camp here for the men, but smoking things and tobacco are running very short for the officers and the soldiers." If only in order to gratify the brave nurse who was mentioned in despatches for her services during the siege of Ladysmith, and who has endured great privations, we are sure that some of our readers will respond to the appeal of Miss Borlase.

### THE NURSING QUESTION IN PARLIAMENT.

LAST week, in the House of Commons, Mr. Lonsdale, who represents Mid-Armagh, asked the Chief Secretary to the Lord Lieutenant of Ireland, with regard to the appointment by the Local Government Board of Ireland of a trained nurse in the Armagh Workhouse instead of a probationer assistant nurse in opposition to the wishes of the local authorities, to explain "why the Local Government Board have not adopted the course offered to them and agreed to by the Guardians of having the legality of the action of the Board, in appointing the nurse contrary to the wishes of the Guardians, and the liability of the Guardians to pay the wages of the nurse appointed under such circumstances, tested in the superior Courts by means of a case stated, and why, instead of adopting that course, a few weeks afterwards, namely, on February 4th, 1901, they issued a general order by which the previously existing power which Boards of Guardians possessed of appointing, with the consent and approval of the Local Government Board, such and so many assistants as Boards of Guardians should deem necessary, has been taken away, and by which Boards of Guardians are now compelled, without regard to their own opinion, to appoint such and so many qualified assistants as the Local Government Board alone shall from time to time think necessary; and whether the word 'qualified' in that order has been given any definition save the words 'such qualifications as the Local Government Board shall think necessary.'" Mr. Wyndham replied as follows:—"The Local Government Board made their general order of February 4th, 1901, because an objection had been raised by the Armagh Guardians to the Board's nursing order of 1895; similar, but not identical, questions arose in other unions. The Board, therefore, decided upon making a general order dealing with the whole subject of the appointment of assistant nurses and attendants, and they were advised that this was the best means of avoiding litigation when irreconcilable differences of opinion on the sufficiency of the nursing staff existed between the Local Government Board and Boards of Guardians. As this course involved the repeal of the previous order referred to by the Guardians, it was not necessary to test its validity in a court of law. The word 'qualified' is correctly interpreted in the question." Mr. Lonsdale has done his duty, and it is not his fault that the question he asked was so wordy that to wade through it is a task. But victory is not on the side of the Armagh Board of Guardians, and we infer that they will now have to submit to the inevitable.

### THE ROYAL NATIONAL PENSION FUND FOR NURSES.

THE brief holiday which the secretary and staff of the Royal National Pension Fund for Nurses enjoyed at Easter must have been welcome, for we hear that, in addition to the exertions and strain of composing the report, its issue produced more letters than Mr. Dick ever remembers on a previous occasion. This is a pleasing proof that it is studied now more carefully than in former days, and the only regret of the secretary is that it is not possible for him to write and thank the nurses individually for the numerous letters he has received from them expressing satisfaction with the report, and gratitude to the Council for their management of the Fund.

### SALISBURY NURSES AND THE PENSION FUND.

AT the annual meeting of the members of the Institution for Trained Nurses at Salisbury it was stated that the affiliation of the Institution to the Royal National Pension Fund for Nurses for the purpose of enabling their nurses to make some provision for the future had worked very satisfactorily. Sixteen of the nurses have joined the scheme in order to secure an annuity of £15 at the age of fifty. The contribution from the Institution amounted this year to £182 15s. 9d., in addition to the sum paid by the nurses. The chairman in his speech said that, "if they could get suitable persons to come to the Home, they would now obtain advantages which they had never had in previous years—as, for instance, the Pension Fund. Fifteen pounds was not a very large amount to secure as an annuity at the age of fifty years, but still it was a useful amount." Another indication of the prosperity of the Institution is that £95 was divided last year amongst the nurses in bonuses.

### THE "SPLENDID TESTIMONIALS" OF A WORKHOUSE INFIRMARY "NURSE."

IT is to be hoped that the patients at the Haverfordwest Workhouse Infirmary do not require much nursing. At a meeting of the Board of Guardians the other day an assistant nurse was appointed. There was only one applicant, and she had sent testimonials from Mr. Davies, of Westfield House, the vicar of Rosemarket, and the schoolmaster of Rosemarket. The chairman said that the testimonials, which were not read, were "splendid ones," though neither of them was from a matron or a doctor. After this remark, the applicant, who is described in a local paper as a smart-looking young woman, was called into the room, and said that she had been living with her cousin for six years and had not been in any situation. Then followed the confession that "she had not had any experience of nursing," but perhaps, with some diffidence, she added that, in spite of this trifling drawback, she thought she could "manage the work." The only comment her statements provoked seems to have been an inconsequent inquiry by the Rev. Peter Phelps whether "she was engaged to be married," and it appears that during the "loud laughter" occasioned by this curious clerical sally, the applicant retired from the room. Subsequently, on a vote being taken, she was appointed assistant nurse. As long as smart-looking young women, with splendid testimonials, and no experience of nursing, are selected by Poor Law Guardians to tend the sick under their care, workhouse infirmary nursing is not likely to attain a proper standard, however thoroughly trained the superintendent nurses may be.

### NURSING AT ST. HELENA.

NURSES who are anxious for foreign experience may like to know that there will be a vacancy shortly in the Civil Hospital at St. Helena. The engagement is made by the Government, and the salary offered is not large, £40 per annum. All expenses are paid out on the understanding that the agreement is to hold good for three years. If the nurse leaves before that time, the money expended on passage, &c., is to be refunded. But, on the other hand, there are many worse and more unpleasant places to work in than St. Helena, and just at present, owing to the presence of so many Boer prisoners, the island possesses a particular interest. The climate is quite healthy, though warm, and the distance from the Cape is only six days' voyage. The matron has been in her present position for six years, the nurse who is leaving in May or June over four, and another worker, now at home, was also at the hospital for some years. Applicants must have had some experience in medical and surgical work, and must not expect in a small Colonial hospital all the up-to-date appliances to be found in England. As the staff is very limited, the matron is anxious to find a lady of congenial tastes, because, as she says herself, "I should like our friends to be her's also."

### THE LOCAL GOVERNMENT BOARD AND THE MATRON OF CROYDON INFIRMARY.

Owing to the fact that we went to press last week a day earlier than usual, our correspondent's report of the decision of the Local Government Board in regard to the application of the Croydon Guardians to appoint a superintendent of nurses did not reach us in time for publication. We now give in another column the list of the duties which the superintendent of nurses is expected to perform. It is not stated whether the list was submitted to the Local Government Board, and, as it is only dated April 2, we cannot believe that the authorities at Whitehall had seen it when they sanctioned the appointment of a superintendent of nurses, since a glance at it shows that the duties are practically those which have hitherto been performed by the matron. Of course, it would be impossible for Miss Julian to continue in office under such conditions, and if the Local Government Board wish to get rid of her we think they ought to say so. There is no possibility of mistaking the intention of the Guardians. They want to force her to resign.

### THE "FRIVOLITY" OF SICK NURSING.

A member of the Forfar County Council having proposed that the sum of £100 should be voted to provide a nurse to teach the people of the county the most approved methods of attending to the sick, it was objected by another member that "this was not a time to spend money on pure frivolity." Nursing has been described in many odd ways, but never before that we are aware of has it been characterised as pure frivolity. It is to the credit of the Forfar County Council that the person who opposed the vote on such extraordinary grounds could not find a seconder, and perhaps when a trained nurse has been at work in the county for some little time, even the single dissentient to her engagement will be ready to admit that the public money has not been thrown away.

### A BEGINNING AT MARCH.

It is pleasing to observe that the town of March has made a move in the right direction. At a meeting of inhabitants in the Guildhall it was decided, after an explanatory speech by Mrs. Walton, M.A., who pointed out that there are now district nurses at work in Peterborough, Whittlesea, Wisbech, Ely, Littleport, Huntingdon, St. Ives, St. Neots, Ramsey, Shelford, Sleaford, Spalding, and Stamford, and expressed her belief that there would be no impossibility in raising an annual sum of £90. With one dissentient, the Rev. G. F. Wells, who seemed to think that because one nurse could not cover the whole ground, it was no good starting an association, the proposals were warmly supported, and £27 was promised in the room towards the amount required. When the advantages of having one fully-trained nurse at work among the poor of March have been once experienced, it will be comparatively easy to extend the operations of the Association, especially if, as a working man happily suggested, a number of his class contribute half-a-crown annually towards it.

### ACTION BY A NURSE.

The necessity for clear and definite business arrangements when making nursing engagements is again illustrated by a recent case at the Bow County Court. A certificated nurse, Miss Mary Frost, claimed £5 13s. 9d. from the East Ham Urban District Council as salary in lieu of notice and travelling expenses. The plaintiff had excellent testimonials from hospitals and doctors, and had been a nurse for thirteen years. She was engaged by the matron of the Diptheritic Hospital about February 13th, and was told to commence her duties on Monday the 25th. Miss Frost, who travelled up from Wisbech in Cambridgeshire, arrived at 8.40 in the evening of that day, did not see the matron, nor was she given a copy of the rules of the institution, but she had her supper and went to bed, being shown her work next day by another nurse. After two and a half hours' work on a very disagreeable task, she was called for by the matron, who reprimanded her for coming so late the previous evening, told her she was no good, and summarily dismissed her. No statement as to the hour at which the nurse was expected by the matron to arrive had been specified, and the matron was not sure whether at the first interview with the plaintiff she had mentioned that she would be engaged only for a month on trial, but she said she had intended to have done so. The matron contended that the nurse virtually discharged herself because she did not like to share a bedroom with two other nurses. Judge French, after hearing the evidence of another nurse, gave judgment for plaintiff for the full amount with costs.

### SHORT ITEMS.

Nursing sisters Dutton and Massey are reported "slightly improved."—At the chapel in Guy's Hospital on Good Friday the Three Hours' Devotion was conducted by the Rev. H. V. S. Eck, vicar of St. Andrew's, Bethnal Green. The service was arranged in consequence of many requests.—The secretary of the Friedenheim Hospital, while mentioning in the financial report for 1900 the receipt of £294 towards the cost of the proposed nurses' home, says, "This is a very urgent need." A building licence, he adds, is now being sought, and "it is hoped that the liberal help of friends will enable us to proceed with our work."—In making an urgent appeal for help towards clearing off the debt of £2,900 still remaining on that excellent institution, the Free Home for the Dying, Clapham Common, which is under the care of the Sisters of St. Margaret's, East Grinstead, the council point out "that the work has greatly increased since the removal to the new building."—At Newcastle-on-Tyne last week Miss Mary Moore, a nurse, obtained £80 damages for breach of promise of marriage against James Burrell, lately bar manager at South Shields.

### Lectures to Nurses on Medicine and Medical Nursing.

By NORMAN DALTON, M.D. London, F.R.C.P., Physician to King's College Hospital.

### LECTURE VIII.—ANÆMIA.

THE word "anæmia" means simply bloodlessness, but there are several varieties to be considered. Some authors divide cases of anæmia into primary cases and secondary cases. When we know the cause of the anæmia we call the case a case of secondary anæmia. For instance, the anæmias due to loss of blood (such as occurs when a large artery is cut or in severe hæmorrhage from the lungs or stomach) and those due to starvation (such as occurs in stricture of the œsophagus or cancer of the stomach) are called secondary anæmias. On the other hand, when we do not know the cause the case is said to be one of primary anæmia. As our knowledge increases, however, it will probably be proved that all cases of anæmia are secondary to some other affection.

Three forms of anæmia will be discussed in this lecture. The first is the form which is so common in young women from the age of 14 to that of 25. In this form the red blood corpuscles are not only less numerous than they ought to be, but, in addition, each corpuscle contains less of the colouring matter of the blood (called hæmoglobin) than it should, so that the total amount of hæmoglobin in the blood is very much diminished. The second form is called "pernicious anæmia," and it is most common in middle-aged men. In this form the red blood corpuscles are reduced in numbers to an extreme degree, and the blood also contains corpuscles that are not found in it in health. The third variety is called "leucocythæmia," and occurs at various ages. In this form the white corpuscles are enormously increased in number, and unnatural white and red corpuscles may be found in the blood.

All cases of anæmia present the following symptoms. The skin, gums, and conjunctives are pale, and the patient usually feels chilly. All the symptoms of progressive asthenia, such as were described in a previous lecture, are present, beginning with a feeling of being easily tired, and going on to extreme weakness, shortness of breath on exertion, and a tendency to faint. Sometimes the feet become œdematous, as in cases of heart disease, and sometimes the face and eyelids are puffy, as in cases of kidney disease. In spite of the weakness the patient is often quite fat. In some cases there is a pain in the stomach whenever food is taken. This pain may bring on vomiting, and it is frequently noticed that when the stomach has been emptied by vomiting the pain passes off. The bowels are usually constipated, and in women menstruation is scanty or ceases altogether. This last symptom often causes needless anxiety, because women think that it is the absence of the menstrual flow which is producing the ill-health; and among the poor I have known elderly women who were anxious to administer concoctions of injurious herbs to young girls with the object of producing a return of the menses. The fact is that the amenorrhœa is due to the anæmia, and the periods soon return when the anæmia is cured.

In the common anæmia of young women the skin is often of a yellowish-green colour, on which account the disease is called "chlorosis." In some of these cases the cheek may be fictitiously red, so that it is necessary to look at the conjunctivæ and gums in order to detect the pallor. The urine is usually pale and abundant. We do not know the true cause of "chlorosis," but it has been attributed to bad food, to working in bad air, to want of sunshine, to the rapid development of the sexual organs, and to poisoning of the blood brought about by chronic constipation. The disease is in most cases rapidly cured by iron and aperients.

In "pernicious anæmia," in addition to the symptoms which are common to all anæmias, very bad attacks of pain in the stomach and vomiting, irrespective of food, occur. These attacks are called "gastric crises." The complexion of the patient is "lemon" coloured and the urine is dark. Further, hæmorrhages may occur from the nose, the stomach, or from any part of the body. Sometimes small hæmorrhages, like flea-bites, are seen in the skin, and frequently the retina is the seat of minute hæmorrhages which impair the sight. Lastly the asthenia is most severe, and serious fainting fits occur, in one of which the patient may die. Some fever may be present from time to time. The cause of pernicious anæmia is probably some poison absorbed into the blood from the intestines. The poison destroys the blood corpuscles.

In "leucocythæmia," which is sometimes called "leukæmia," the symptoms common to all varieties of anæmia are also present; and hæmorrhages and fainting attacks such as occur in pernicious anæmia are met with, but gastric crises are not so common. The skin is dull-white in colour. The temperature is often high for days together, and the fever is usually of a remittent type; that is, it may be 102° or 103° at one time of the day and 100° at another time. In one form of leucocythæmia the spleen becomes large, and it is sometimes so very large as to make the whole of the abdomen prominent.

Cases of pernicious anæmia and of leucocythæmia are usually fatal in a few months or years. They are often relieved or apparently cured by the administration of arsenic.

It is only the severe forms of anæmia which come under the care of the nurse. They require to be most carefully fed, to be protected against cold and against exertion. Sudden movements are particularly injurious, because they may bring on a fainting attack; but the easiest occupation, such as knitting, may be too exhausting if carried on for a long time. The temperature chart must be religiously kept, and it is advisable, in addition to taking the morning and evening temperature, to take it at odd times on different days, so as to detect irregular rises. When possible, the patient should be guarded against anything likely to excite the emotions. Once dropsy has set in every care must be taken to protect the dropsical part from injury, as the slightest abrasion may become the seat of septic inflammation.

The slightest sign of bleeding from the nose, or of blood in the expectoration or in the stools, &c., or under the skin should be carefully noted. The gastric crises also call for careful observation, as they usually occur in the doctor's absence. Nurses should note (1) how long after food the pains came on; (2) the kind of food last taken and the quantity; (3) how much time elapsed between the onset of the pain and the occurrence of vomiting; (4) whether the vomiting relieved the pain. Any remarks which the patient may make as regards the kind of pain or the feeling of nausea should be noted, and the vomited matter should be reserved for the doctor's inspection in order that he may know how far the food taken has been digested. If the attack occurs suddenly, and the nurse have no instructions as to its treatment, she may apply hot fomentations or a poultice to the pit of the stomach, and fifteen drops of laudanum may be put upon the flannels unless the patient be greatly debilitated. Drinking warm water may relieve these cases; but unless the patient has tried this remedy during a previous attack a nurse should not recommend it on her own responsibility.

### Nursing at the Great Yarmouth General Hospital.

By a Correspondent.

The other day I called upon the Matron of the Great Yarmouth Hospital, who received me with the inquiry:

"It is especially about the nursing that you wish to hear, is it not?"

"Yes," I replied, "it is, for I understand that you have successfully reorganised the nursing staff, and that the new arrangements work most satisfactorily."

"The staff," said Miss Diver, "certainly is improved, and I am, so far, very pleased with the result of the new state of things. But before I go fully into this question, would you not like to see over the hospital while it is still daylight?"

The Wards.

I assented, and we walked out of her sitting-room, down the corridor, and into one of the men's wards. It was very bright and comfortable looking.

"How many beds have you altogether in the hospital?" I asked.

"Sixty, and we have four large and two small wards."

Passing out of the first ward we came into another—a women's ward. We looked round it, and then the matron said: "I want to show you a special feature of our hospital—the rooms for convalescents. Let us go upstairs. Here is one"—and she threw open the door of a large and most cheerful room where several patients were sitting. "They have a small library, you see," she continued, "and games and many other diversions to while away the tedious hours of convalescence. I consider these rooms of the greatest value, and cannot imagine what we should do without them now. We have one for the men and one for the women."

Next we saw the remaining wards. Those on the first floor are particularly cheerful and have from their windows a good view of a busy part of the town. We passed through these wards and into the theatre.

Here the matron observed: "Though we pride ourselves upon our surgical work—and we certainly get splendid cases—our operating theatre still leaves much to be desired. But I hope that we may have a new one before long."

The Nursing Staff.

When we returned to the matron's own room I said:—

"And now, please, I want to ask you all about the nursing. Of what does your staff consist?"

"Three sisters and eight probationers. We used to have assistant nurses when I came first here—six years ago—but the plan did not answer, and I find our present arrangement a great improvement in every way."

"Have the probationers a regular three years' training?"

"Oh, yes. That regulation is in full swing now and answers admirably."

"Have they lectures and examinations?"

"They have lectures from the resident house-surgeon—who is so particularly conscientious about teaching and lecturing them—who also examines the nurses. The visiting staff, too, examine the probationers. The nurses get an excellent surgical training, and I may say that we are very up-to-date in our surgical work. A former house-surgeon—Mr. R. W. Monsarrat—took a great interest in the nurses' work and most kindly drew up a list of rules for them. They supply a great want. It is a considerable help for a new probationer to have such rules in print, and prevents the slightest confusion at operations."

Rules for Operations.

"It might be interesting to mention some of them."

"I will explain how the principles are carried into effect. Four nurses are present at ordinary operations, one to the right and two to the left of the table, the fourth near the left hand of the anæsthetist. They take the most complete precautions. Their aprons are clean, their cuffs are removed, and their sleeves are turned up to the elbow. Their hands are washed energetically with soap and water for five minutes, special attention being paid to the nails; then they saturate the epidermis with turpentine and wash and rub for another two minutes with a solution of biniodide of mercury (1-1000). Each nurse attends absolutely to her own duties alone. One has charge of the instruments, sutures and ligatures, and touches nothing else after disinfecting her hands. Another stands to the left of the foot of the operating table and is responsible for the sponges or swabs which may be required; a third receives the soiled sponges from the surgeon in a surgically clean lotion bowl and rinses them thoroughly in warm boiled water and soda. If swabs are used she is responsible for their supply. It is her duty to arrange the carbolic towels. The duties of the fourth nurse vary with the occasion. She alone touches the blanket when the site of operation is uncovered; it is her duty to surround the area of operation with mackintoshes: she removes the bandages, if required to do so by the surgeon. She removes any instrument, sponge, towel, &c., which may happen to fall on the floor. She alone goes to the instrument cupboard or carries any message from the theatre if required to do so by the surgeon. The operating surgeon's wants are as much as possible anticipated, and his orders obeyed quietly and without hurry. If there is any difficulty the nurse must refer to the house surgeon."

The Probationers.

"Have you much difficulty in obtaining probationers?"

"In getting really suitable ones—yes. But now that the training is becoming known and valued this trouble will, I hope, no longer exist. At present the system is almost in its infancy, but it is thoroughly started; and I have every confidence that future developments will firmly establish and recognise its status."

"Are the nurses who have had three years' training here able to take good posts afterwards?"

"They seem to have no difficulty whatever, though it is chiefly private nursing that they go in for when their time here is finished. Of course they are not able to obtain those appointments which necessitate training in a hospital containing not less than 100 beds."

"Have the probationers to pay any premium?"

"No. They pay nothing. Neither do we give them any salary or uniform. But at the end of their three years they receive a bonus of £10 each."

Hours of Duty.

"What are the hours of duty daily?"

"For the six-day probationers the hours are from 7.30 A.M. to 8.30 P.M., with two hours off duty each day. The two night probationers are on duty from 8.30 P.M. to 8.30 A.M.; and they get from 9 A.M. (after their breakfast) to 12.30 P.M (when they go to bed) off duty every day."

"Have you a night sister, and for how long at a time are the nurses on night duty?"

"One of our three sisters is the night sister. The probationers are not on night duty for more than three months at a time, generally for less—sometimes for much less."

"What is the minimum age for new probationers?"

"Twenty; I never care to take them under that age," said the matron.

Holidays.

"Do you mind telling me something about the nurses' holidays?"

"They have one whole day a month and a fortnight annually."

"Do your nurses enjoy good health on the whole?"

"Excellent! We seldom have any illness among the members of the staff. I expect the splendid air of Yarmouth accounts, in a great measure, for this."

It was easy to see the personal interest Miss Diver takes in her nurses. Most admirable work is done within the walls of this hospital (which was rebuilt in 1887), both with regard to the nursing of patients and also in respect to the teaching of probationers; and it should earn no small reputation as a training school.

As I rose to leave, I said, "Has any nurse from this hospital gone to the front?"

"Yes, we were represented," replied the matron. "One ex-sister, Miss Whiteman, went to South Africa as an Army Reserve nurse, but she was not trained here."

### Through an Epidemic.

BY A LATE QUEEN'S NURSE.

I WAS working as a Queen's Nurse in a town in South Wales. I had been very busy all the hot weather, and had not been able to take my holiday until late; the summer was almost gone, and it was at last arranged that I was to have the three weeks owing to me in September. Week after week some urgent case had kept me on. As soon as one got better another got worse, and as I was the only nurse in a town of 11,000 inhabitants, it stands to reason there was much work. The town was never free from typhoid, and I had nursed already between twenty and thirty cases. I settled my plans, and even fixed on my train, but the week before I was to start business began. "Would the nurse call at Mrs. Jones's: typhoid"—so the brief note was worded from the parish doctor. In less than a week I was visiting ten cases, and I felt I could not take my holiday—it would have looked so like fear; therefore my sister, who was staying with me, went to London for a month and I settled down to work.

### CAUSE AND EFFECT.

The epidemic closely followed the route of the canal which runs through the valley. I had several cases on the upper banks, and very quickly on the lower banks also. I always gave careful instructions to the friends of patients emphasising the importance of not emptying anything into the canal, but it was invariably done, and children would drink of the filthy stream. It was a real case of cause and effect. In one house of five rooms a family of twelve lived; nine of them had it in turn. The mother died, and one boy nearly lost his life through over-eating and drinking raw brandy. After all had recovered, my first patient in the house developed scarlet fever, so abruptly ending my visits, which had been daily for four months. In another house in the same street a mother and child had typhoid severely. Nearly all the cases had boils prior to convalescence. One patient was quite alone, but for her husband, so she had an elderly woman to do for her, who drank the brandy out of the bottle, even before the patient's face, and was nearly always drunk. She used often to wash the bedding and the cups and saucers in the same tub; yet, strange to say, the husband did not get the fever. I visited the patient twice daily for seven weeks, and all that time no one else would go near her.

### A LITTLE "JOB."

In another house in the same street there were two children in one bed; one died. I was not visiting there, as the doctor thought that the mother did well enough.

In another house in the same street a little boy had the most fearful boils. I never saw a case like it; he was covered all over the head, face, chest, and back, but not on the limbs. He was a veritable Job—not for patience, but for boils.

The dirt in some of the houses was indescribable.

### FOURTEEN CASES IN ONE STREET.

In one particular street there were no fewer than fourteen cases. I attended eleven, and four of these were in one house. The first case, the son of a rag merchant, was a boy: he had cellulitis in the neck, and nearly died from suffocation, but recovered. There were a husband and wife together; the man also had a poisoned finger, from which I removed dead bone. The wife had pneumonia afterwards. Another was a young man; he was apparently dying when I went first, but, after I cleansed his clogged-up mouth, he rapidly became convalescent. He disliked anyone but me to comb his hair, for I curled it. The four cases in the one house were sent in as influenza. The eldest boy was very much afraid of the disease, and, when he sickened, used to eagerly scan the chart as soon as I had left. But in a week he suddenly died of perforation. The poor mother was distracted. Another boy had it, but not severely, and a little beyond a woman had it slightly. She was the daughter of the gravedigger. She told me her father always asked if I had been visiting when he dug a grave; if not, he said, "No wonder you want me. I have never to dig graves for nurse's patients!" Could praise be greater?

### FEVER AND SWEETS.

One amusing patient was an Italian of 19, black-haired and swarthy. He was a vendor of ice-cream; his father a potato-chip man. Saul, my patient, believed he got typhoid from a "blow out" of ice cream at Swansea. He used to sell wafers with his creams, and they were carefully kept (in tins) in his room, a small room laden up with goods, and a window very difficult to open. Still, it was a clean house compared to many. I was often offered some wafers after I had finished my work, which I always declined.

There were many cases I only heard of, but could not go to. Two little boys died, who were both known to have imbibed the canal water, and the mother sold sweets, shown in the window just where the bed was—to be handy.

### MEMORABLE DAYS.

At this time the epidemic was at its worst. I had fifteen typhoid cases on my list; in many I could only call in and give instructions, and cleanse the mouth (a thing never readily done); in one very dirty part, called the Green, there were seven or eight cases.

There are some days that stand out in one's memory, and I well remember Sunday, October 1st. It was a very wet and stormy day, and difficult to get about; yet I was harder at work than any other—nine hours. I was sent to two typhoid cases, a boy and a young man, the latter almost dying; but he ultimately made a good recovery.

### OUTSIDE MY DISTRICT.

Then I went outside my district to three cases. I could not undertake them, but went merely to cleanse their mouths; they were all in one house, and they all made good recoveries. I went to a very nice house, where the mother had it badly. She had severe complications, and soon died. I asked the curate to visit one of the patients; he was honest enough to own to fear. I said, "I go there twice a day," and he went too after that. Then came a lull, and my sister having returned, in the middle of November I went away and she undertook my duties. All were now convalescent, and there had been no new cases for about ten days. So I had three weeks' holiday in London.

### FIVE CASES IN ONE HOUSE.

When I returned, my sister told me she had been very busy: she had had ten new cases of typhoid, and several deaths from pneumonia, which now became the general complication. One was the mother of a child whom we had nursed together, and had fed by force because she refused food. The child recovered, but the mother took the fever and very quickly died. Another was a bad and neglected case; the girl (about 25) had a weak heart, and the doctor had ordered her not to be moved. She was not moved for a fortnight, until my sister went, and, with the doctor's help, the task was accomplished. There was a large bedsore, horrible odours, maggots and lice galore, and these people were in a superior position! There were five cases in that one house of four rooms. A brother and sister of twenty-five and twenty-seven laid in one room, with only just room to move between the beds; yet they all recovered.

### A Healer of Ulcerated Legs.

I soon had several new cases of my own, and one was a man who had been very ill, but was now recovering; another man was a "professor" who healed ulcerated legs, his complication was D.T., and he was ill for three months. Then I began to be alarmed at my sister. She seemed very poorly, but whenever I said anything about her health she said I had typhoid on the brain. But about a fortnight after my return she was not able to move about, sitting all day by the fire, and when I took her temperature I found it was high. She had contracted typhoid. She had certainly taken it from the girl with the bedsore. I was in despair. I was visiting a woman and child who were both bad, but I had to give them up; I could not bear the strain, being away from my sister. Fortunately she had it favourably, without complications, and only one small boil; but I felt that I had exposed her to it. It seemed strange: I had nursed seventy cases and she only ten. Yet I did not take it. She was almost convalescent by Christmas, but not taking food. On Christmas day I had a change of cases. A terrible burning accident took place, the woman was burnt all over, and mercifully died of the shock; she had prepared her Christmas dinner overnight, and said they would "have a long sleep to-morrow." She died at midday. It was a terrible Christmas day—one I shall not soon forget. A few straggling cases kept turning up. One was a young man who had had hæmorrhage twelve hours before.

Another case was a little girl who had been ordered sponging, but the aunt who was nursing her refused to do it because it was mid-winter; so I had to go and do it. The child recovered.

### The Gratitude of the People.

Few people realised how bad an epidemic it was. I have not mentioned several minor cases, but the chief ones. No one seemed to think much of it, but it was a really terrible time, and I never wish to work through another such experience. The gratitude of the people was wonderful, and they made me offerings of almost every sort, whatever they worked at. Everywhere I went I was told that but for me they would have died, as no one would enter the house if they knew the fever was there. The work was extremely hard, depressing, and often loathsome; still, I can always feel proud of having done my best to help in troublous times.

### Croydon Infirmary Crisis.

At the meeting of the Croydon Board of Guardians last week a formal letter was read sanctioning, by the Local Government Board, the application of the Guardians to appoint a superintendent of nurses. The Local Government Board made no comment on the general facts in the dispute between the Guardians and the matron of the infirmary.

The following duties of the new "superintendent of nurses" prepared by the special sub-committee appointed for the purpose, were then submitted to the Board for approval:—

1. To undertake the immediate supervision of the nurses, and to enforce punctuality and the due observance of the rules prescribed by the Board for their guidance and discipline.
2. To undertake the training of the probationer nurses and to periodically lecture to them. The lectures to the probationer nurses to be so arranged that a lecture to each of the first, second, and third year probationers shall be given once a week.
3. To be responsible for the nursing in the wards, and to see that the sisters and nurses carry out the orders of the medical officers in charge faithfully and punctually.
4. To visit the wards at frequent intervals, and not less than twice during any day to supervise the nurses in the performance of their duties.
5. To arrange for the disposition of the nurses in the various wards, subject to the approval of the medical superintendent.
6. To arrange for the daily leave of absence of the nurses and to submit the same to the medical superintendent each morning for approval, and when approved to issue the necessary passes.
7. In conjunction with the home sister to preside at the various messes of the nurses, and to take her meals with them.
8. To report at once to the medical superintendent any case of sickness among the nurses, and take care that every attention is shown to those who may be invalided.
9. To keep a report book, in which shall be entered all matters requiring attention, and submit the same to the medical superintendent every morning.
10. To make a nightly round of the wards alternately with the home sister.
11. Not to be absent from the building without the knowledge of the medical superintendent, and having first obtained his permission to do so.
12. To be entitled to three weeks' leave of absence annually.

April 2nd, 1901.

### Appointments.

Bradford Royal Infirmary.—Miss Caroline L. Jenkins has been appointed home sister. She was trained at Bradford Royal Infirmary, where she has since been sister and night superintendent.

Chelsea Infirmary.—Miss Marian Cotts has been appointed charge nurse. She was trained and certificated at University College Hospital, Gower Street, where she has since held the post of staff nurse.

Dartmouth Cottage Hospital.—Mrs. C. Kaye has been appointed house matron. She was trained at the Royal Infirmary, Liverpool, and has since been sister of male wards, and of female and children's wards at the General Hospital, Rotherham.

Fever Hospital, Gore Farm, Darenth.—Miss Margaret Jones has been appointed temporary matron. She was trained at St. Bartholomew's Hospital, and has subsequently held the posts of night sister at the Incurable Hospital, Leamington; charge nurse, night superintendent, housekeeper, and assistant matron at the M.A.B. Northern Fever Hospital, Winchmore Hill.

### Presentations.

Royal Infirmary, Hull.—Miss Mary Bayldon on leaving the Royal Infirmary, Hull, has been presented by the nursing staff with a Queen Anne silver teapot, cream jug, and sugar basin. Miss Bayldon has been on the staff for nine years, and for the last eighteen months has held the post of night superintendent. Her departure is much regretted by all.

### Death in Our Ranks.

Nurse Ethel Brooks died at the East London Hospital for Children, Shadwell, from enteric fever on Wednesday, April 3rd. She had been a probationer since September 1899, and was only 21 years of age.

### Lectures to Head Sisters.

BY MISS E. MARGARET FOX, MATRON OF TOTTENHAM HOSPITAL.

### LECTURE I.—A HEAD SISTER'S DUTIES TOWARDS HER PROBATIONERS.

(*Continued from page* 6.)

YOU should also exercise a little supervision in the matter of the appearance and manners of your probationers. Show a new one how the cap should be put on, how the uniform should be worn, how they are expected to wear their hair and fasten their collars. Point out to them that the hospital rule which forbids untidy hair flying in all directions has a certain amount of sound common sense at the back of it, for if kept within reasonable limits the hair is less likely to catch up dirt and certain objectionable living things. Of course, this implies a careful attention on your own part to personal neatness. If a newcomer sees her head sister going about with untidy hair, cap askew, dirty cuffs, and crumpled collar, with various stains of picric acid, ink, or iodine on her dress and apron, how can she either respect her or attend to what she says? And if, in addition, the head sister is noisy, wears shoes with clattering heels, thinks it does not matter how she treats her dress because it is "only uniform," if she is careless, unpunctual, untidy, what attention can she reasonably expect when she impresses on her probationers the necessity of being tidy, or quiet, or reproves them for being late on duty?

Good hospital manners must be enforced in every well conducted ward. Without giving yourselves any foolish airs, you must exact a certain amount of ceremony from your probationers, not allowing them to remain seated or to lounge when you are giving them orders, or when any visitors, doctors, or hospital authorities are in the ward. Keep up the official titles of "sister" and "nurse" when on duty, and do not address one another by your Christian or surnames in the hearing of the patients. Although it seems only a trifle anything like that weakens your authority, and makes it more difficult for you to maintain discipline. Do not talk to your probationers too much. The less talking that goes on while on duty the better. A group of nurses, chattering in the ward kitchen, is a great nuisance to the patients in the ward, who are sure to feel themselves neglected if no one is at hand to attend to them. Be quiet yourself, and others will instinctively be quiet also. Give your probationers an encouraging word from time to time, for it is so easy to get discouraged when one is new to the work, and of course there is no need, while avoiding useless chatter, to do everything in a stony silence. Give few orders, and make few rules, but do see that those orders are carried out and those rules obeyed, or you had far better make none at all. Learn to discriminate between a new-comer's genuine forgetfulness of an order and the wilful or careless neglect of one from a probationer of longer standing. In the first case, give the forgetful one a kindly reminder, but in the second, do not be too ready to accept the inevitable "I forgot." Neither must you perform, nor allow someone else to perform, the neglected duty unless it is imperative it should be done at once.

If it is anything that can be left a few minutes, fetch the probationer back again to do it, no matter if she has just gone off duty. She may think at the time that you are very hard on her, but she will thank you for it afterwards. Do not allow her to answer you back, or to argue with you about any order you may give her. At once make it clearly understood that you will not have it, but take care to say that and all other reproof in a purely official manner, and beware of losing your temper or allowing any personal feeling to appear. Your wards must be ruled by kindness and moral suasion largely, and remember you are dealing mostly with conscientious and intelligent women, who, like yourselves, are anxious to do the best possible for the patients. With the exercise of courtesy, tact and sympathy, combined with firmness and capability, it is generally possible to manage a ward without friction. Of course, it is idle to expect things always to go smoothly. Trouble will occasionally arise—people will be difficult to manage, and be careless and forgetful.

But do not be always fault-finding. It is dispiriting, and apt to bring out the worst side of people, though you must not let those under you become slack. Praise, as well as blame, when you can honestly do so. A little appreciation of one's efforts is very cheering, and we have no right to make life any harder for each other than we can help. When you must find fault with your probationers, do not of choice do so in the presence of the patients; it is an unnecessary humiliation. Speak to them quietly, by themselves, and if after a reasonably gentle reproof you find the offence repeated, warn them that you will be obliged to report them, should it occur any more.

Of course you will not fall into the error of reporting your probationers for every trifling fault. It shows a certain acknowledged weakness of character to have to appeal constantly for confirmation of one's authority to someone else. There is in it an echo of the child's futile cry—"I'll tell my mother of you!" or of the weak-minded parent who threatens his boy with the schoolmaster's cane, instead of using his own authority. Deal with the minor difficulties of everyday life promptly, as they arise, and cultivate self-reliance. But in any graver matter—and alas! even in hospitals, such a contingency will very occasionally occur—do not, from a mistaken sense of kindness to the delinquent, hesitate to report it at once. It is the truest kindness to everybody concerned to be firm in putting down what is really wrong; and while "telling tales" is despicable, there is a well-marked distinction between that and the reporting of serious faults or mistakes.

Official report giving too, is quite another thing, and every sister should be prepared to give that correctly of each probationer who passes through her ward. Various questions as to her character and capabilities are then asked, and you should strive to answer them, not in a casual manner, but thoughtfully, with a view to the probationer's ultimate success. If you have not found her careful or obedient, do not hesitate to say so. A few words of caution before entering another ward may supply the necessary filip to exertion. She may succeed better under another sister, and with a different set of patients. It is no kindness to conceal her defects. We are none of us perfect and we are here to learn. If your probationer is not willing to be taught, it is of no use her staying. Report her progress faithfully so far as you know it, without concealment or evasion. Do not let a careless good nature blind you to a strict sense of right. It is a duty you owe to her and to your matron, who has far less opportunity of judging her than you who work alongside her all day long; she is obliged to let her opinion of the probationers be biassed largely by yours. So try to make your reports as accurate as you know how.

It is very hard to be just—how hard you all know, when you sit down to write your probationers' reports—and long to slur over the culpable carelessness of that nice, bright girl you cannot help liking, and to be fully alive to the methodical punctuality and rigid conscientiousness of that other, whose voice and manner grates on you all day long. It is so much easier to be unselfish, kind, easy-going, indulgent. Perhaps, because it is so hard to be just and yet so necessary, that it is placed first of all in the list of Divine requirements from man. "To do justly" comes before "to love mercy." The rock of favouritism is one on which many an otherwise good sister has come to grief. It is absolutely fatal to the successful working of a ward. It demoralises all round. Of course we cannot help having preferences, nor control altogether our affections, but we can control our manifestations of them. Favouritism weakens your authority, undermines your influence for good on the others, and spoils the favoured one who gets disliked in consequence by the other probationers, and it makes the task of training her much harder for the next sister under whom she works. If you aim at being really good ward sisters, you will avoid making favourites among your probationers.

### The International Congress at Buffalo, New York.

By Maud Banfield,

General Secretary of the Congress; Superintendent Polyclinic Hospital, Philadelphia; Lecturer in Hospital Economics at the Teachers' College, Columbia University, New York; Member of American Superintendents' Association; American Association Training School Superintendents, M.R.B.N.A., &c.

To the International Congress of Nurses—which will be held in Buffalo, one of the lake cities of New York State, U.S.A., during the third week of next September—delegates and visitors are expected from England, Scotland, Ireland, Cuba, South America, and even far-off Australia, whilst letters of encouragement and sympathy have been received from prominent members of the profession in Germany, Italy, Switzerland, South Africa, Denmark, Holland, India, Ceylon, Japan, and New Zealand, regretting lack of time and means, but in many cases offering papers, and always expressing heartiest sympathy.

### The Programme.

The programme will be divided into sections, and the papers read will, as nearly as possible, be by women who are or have been actually engaged in the special branch of work referred to. In this way it will be sought to make the congress of much practical utility, and the publication committee hope eventually to present as complete a picture as possible of present conditions, and ideals for the future. The initial subject will be "Hospital Administration in the United Kingdom, the Colonies, Abroad, and in the United States." The absolutely different basis of government prevailing in different countries should bring out some interesting and instructive facts. The relation of the governing bodies of hospitals to nurse-training schools concerns us all, and will no doubt be ably dealt with. The desirability, or otherwise, of women trained and untrained on hospital boards will be referred to. The educational section should prove of paramount interest. The comparatively recent attempt at preliminary teaching before the nurse enters the hospital and the finishing touch as instanced in the course at Columbia University, New York, are amongst some of the practical points which will, no doubt, arouse energetic discussion. District nursing, "nurses' settlements," nurses on boards of health, hourly nursing, army, navy, and Red Cross nursing, and organisation, are amongst the many subjects on the programme.

### The Associations.

In America the nursing profession is fortunately rent by no schisms within its ranks, although individual heretics who care only for earning their own dollar are as prevalent here as elsewhere. The associations are composed exclusively of nurses; medical men or women and trustees of hospitals are admitted to meetings as visitors only at request of members, for in time we hope to educate them to a sense of the duties and responsibilities of their position; and although we often differ from each other with great vigour, and express our sentiments with a frankness and energy which I imagine an outsider would not consider altogether parliamentary, our sense of humour, or some other sense, prevents our thinking or acting as if there were no salvation for the other fellow. We feel that much is to be learnt from these meetings, and the members often travel hundreds of miles to attend the annual Convention.

### Suggestions to Intending Visitors.

All nurses will be welcomed at Buffalo next September, whether they come as delegates or as private individuals Members of the reception committee, distinguished by a badge or cap to be decided on later, will meet all visitors, and take them to their rooms, which will be previously engaged if desired. Intending visitors should write to Miss Damer, 55 Mohawk Street, Buffalo, New York State, and as Buffalo will be very full, this should be done as soon as possible. Prices will range from 4s. (a dollar) a day for room, and 1s. for breakfast. The dollar-room rates, however, mean two in one room. The Woman's Christian Association is building a lodge just outside the Exposition gates at which the price is to be two dollars (8s. 4d.) a day, including meals. This is very reasonable, and it will probably be necessary to engage these rooms a long while beforehand. The American newspapers say that the Buffalo Exposition is to be even more beautiful than the World's Fair, although not quite as large. Over 300 acres will, however, probably provide us with enough ground to cover at the end of our sessions. Niagara Falls are less than half an hour by rail from Buffalo, and a very delightful trip might be arranged by a nurse who has six weeks' holiday, landing in New York or Philadelphia, a railway journey through the Allegheny Mountains (the "Switzerland of America") to Buffalo, visiting Niagara and Toronto, steamer through the 1,000 islands of the St. Lawrence, a day or so in Montreal and Quebec, and thence direct to Liverpool. The cost of railway and steamer fares this side for the trip would be as nearly as possible £5. Other expenses would necessarily depend on the taste or the purse of the traveller. If it were possible to invest a little more time and money this journey might be extended so as to include visits to Philadelphia, Baltimore and Washington, for the sum of £12. Steamer rates vary with the line and the accommodation, starting with £7, £8 and £10 on the Philadelphia line to New York but steamship companies are apparently not willing to reduce their rates for parties of less than 100. Any further information in regard to journeys, time, necessary clothing, &c. (which may be very simple), will be willingly afforded.

### Wants and Workers.

Wanted.—Water bed for poor invalid nurse. Address Mrs. Bretland Farmer, Claremont House, Wimbledon Common.

### To Nurses.

**We invite contributions from any of our readers, and shall be glad to pay for "Notes on News from the Nursing World," or for articles describing nursing experiences, or dealing with any nursing question from an original point of view. The minimum payment for contributions is 5s., but we welcome interesting contributions of a column, or a page, in length. It may be added that notices of entertainments, presentations, and deaths are not paid for, but, of course, we are always glad to receive them. All rejected manuscripts are returned in due course, and all payments for manuscripts used are made as early as possible after the beginning of each quarter.**

### Echoes from the Outside World.

### AN OPEN LETTER TO A HOSPITAL NURSE.

ALTHOUGH the newspapers for our benefit frequently paint word pictures of the condition of South Africa at present, it is only when one gets a private letter that one really grasps the sad state of the poor desolate land. A private correspondent writing by the last mail from a town in the Orange River Colony which was originally a famous resort for delicate people, because of its health-giving air, says: "It is so sad now to see our plague-stricken little town, which has always borne the enviable reputation of being one of the most salubrious spots in South Africa. This outbreak of enteric is, I know, one of war's scourges, but it is none the less fearful. There is hardly a household without its patient, and scarcely a nurse to be had. For the past two months I have been busy, night and day, tending a great friend, who had fallen a victim to the epidemic. The family had no one to assist them, and when the girl succumbed she had been nursing her brother, who was ill of the same disease, for a couple of weeks. I am keeping well myself, which, all things considered, is wonderful. The thousands of cattle, sheep and horses quartered in and around the town—loot stock which has to be protected—are in themselves enough to breed any disease. The poor soldiers have died in great numbers. One day lately there were six funerals, two days after there were two more. The hospitals, I am thankful to say, have now been moved out of the town. Tents and small forts are to be seen from all the surrounding kopjes. Pickets are stationed three or four miles from the town, and every night flashings are to be seen from the different camps. No one is allowed to go beyond the town lands without a pass, lights are supposed to be out after 10 P.M., but free passes are constantly given, always in cases of sickness or entertainments, of which there are very few."

"THE soldiers come to church with their rifles and cartridge belts, so we do not forget this is an enemy's country although all is fairly quiet. Boers are continually sniping, and stealing cattle and horses from off the very townlands in spite of the protectors around us. The first volunteer corps of the O.R.C. has been formed here; they have been called out two or three times. Yesterday when they turned out they had quite a military appearance as they went out to chase supposed Boers. The idea is that they are only here to protect the town and its environments; but they generally go further afield. The misery brought upon all in the O.R.C. one would think should force the Boers to surrender; but still they hold out, and, unfortunately, British and Boers are equal sufferers. We are all too weary to read the papers unless it is something very out-of-the-way, and the poor soldiers are more deadly tired of it all than we are. It is heartrending to see the quantity of stock which must die from want of food when the winter comes, and how the country is already suffering from the lack of any cultivation. The famine will of course be worse yet, and we hardly dare to look ahead for fear of the spread of the bubonic plague. If it becomes very bad in Capetown the inhabitants and refugees will rush inland, and of course bring the disease with them.

THE fact that April 17th, the date fixed for the end of the general mourning, comes so soon after Easter evidently decided a few undecided persons to forestall the Royal command by a week or two, for an immense number of people took a final farewell of their mourning last Sunday. I suppose it is because our eyes have become so thoroughly accustomed to black that the colours just now appear unusually vivid, but several of the royal blue, cornflower blue, or china blue coats and skirts which I met almost dazzled me by their brilliant hues, and seemed wanting in refinement. But this impression will probably soon wear away, and we shall even look with favourable eyes upon the gold-bedizened materials, feathers and flowers which are to be the rage of the coming season. Triple skirts also are to be worn—two out of the number being, of course, only simulated. One of the most successful of these arrangements I will quote, as it may be useful to some of you. The wearer confided to me that she had been obliged to have a new black dress upon the Queen's death, and could not afford another spring garment as well. So she spent a little extra on the "doing up" of the one already purchased, and really it looked exceedingly nice. In place of the black frills which had originally trimmed the bottom of the skirt, two simulated skirts were added, one of very dark scarlet, one of dark green, the third and top skirt being the original black. Both the red and green skirts were softened at the edge by narrow black fancy braid. The sleeves were treated in the same triple manner at the wrist, and the collar was to match. Also beneath the black bolero the red and green peeped out in the same manner as at the bottom of the skirt. A black toque with red rosettes and green wings completed a neat and cheap yet stylish early spring costume.

FOR some time past the island of Jamaica has been in a bad way, and one has heard sad accounts of the want and suffering caused by the failure of the sugar cultivation. But now it seems likely that, although fresh life cannot be put into the original industry, a new source of money-making has been opened. The climate, according to a doctor who has just come over, is exceedingly good for consumptives. Twenty-five years ago he went to Jamaica almost a hopeless invalid, since then he has become so strong that he has walked across Africa from the west coast to the east. But it has yet to be proved that what cures one man will cure another, and meanwhile the fast-steaming vessels that have lately been started are bringing supplies of fruit to England for which a ready market is found. The fruit, of course, has always been abundant in Jamaica, but all attempts to convey it to England have failed because the voyage was too lengthy to allow of the cargo arriving unspoilt. Now, however, faster vessels and good cool storerooms (the rooms are never allowed to become really cold, because a touch of frost or ice naturally injures the fruit) have removed the obstacles, and both oranges and bananas are coming over in splendid oondition. I saw some of the oranges the other day. They are a wonderful size, almost as large as the head of a new born baby, and somewhat the same bullet shape. They are not as yet very cheap, but the bananas are exceedingly moderate, and will probably cause this popular fruit to become yet more popular. But like many other blessings, this is not unmixed. The street urchins purchase and eat, and heedlessly throw the skin on the ground as they walk along, with the result that a large number of serious accidents have already occurred. The banana refuse is exceedingly slippery, and the warning issued by the Chief Commissioner of the London Police upon the subject is well timed. Moreover, the ordinary woman as she walks abroad can do something to help if she treats a banana skin in the same manner as I hope she does a piece of orange peel, namely, pauses one second to kick it off the pavement into the gutter.

IT must sometimes have been the experience of a few of my readers to find that a patient after recovery from a serious illness is left partially deaf. To such sufferers a little book written by Miss Isabel Pollock, daughter of the eminent judge, must be distinctly cheering. The lady herself lost her hearing through illness, and experienced, in her own person, the tedium of continual silence, and the pain of feeling "left out," but now has recovered in a large measure happiness for herself and her friends by the study of lip reading. She maintains that two dozen lessons will teach a person of average intelligence who has only become deaf from accident, not from childhood, to lip-read quite easily, and dwells on several instances of success within her own knowledge. The names of a few male and female teachers in various parts of the country are added at the end of the little book, which is entitled "Lip Reading," and is published by Simpkin, Marshall and Co., Paternoster Row, at a trifling cost.

### Everybody's Opinion.

[Correspondence on all subjects is invited, but we cannot in any way be responsible for the opinions expressed by our correspondents. No communication can be entertained if the name and address of the correspondent is not given, as a guarantee of good faith but not necessarily for publication, or unless one side of the paper only is written on.]

### PRESENTS TO NURSES.

"Nurse Prudence" writes: As a nurse of ten years' experience of private nursing I must say I do not agree with "Nurse's" remarks regarding the giving of presents to nurses. I have had presents made to me at different times and have taken them as a kindly acknowledgment and showing an appreciation for services rendered. I see no reason at all why any nurse should object to a patient giving her a present above her fee, any more than a vicar or curate who receives a purse of money from his parishioners as a testimonial showing their appreciation of his services. I do not think a nurse loses any respect by accepting a present, but by refusing it she would undoubtedly hurt the feelings of the giver and probably lose a friend. The editor has dealt with the subject in a reasonable way, and I believe the majority of commonsense nurses will agree with him.

### THE NURSES' CO-OPERATION.

"A Mental Nurse" writes: As one of the staff of the Nurses' Co-operation I feel bound to raise a protest in defence of the home. I would like to know how such a home and club would work without rules? We have freedom when we go to the theatre, we have a pass-key, &c., and I have always found the matron and assistant most amiable. Those nurses who have no home in London know very well that it is much cheaper going to the home than paying rent all the year round. I myself sold my furniture last August for I was hardly ever in my room, and have derived great benefit from the home in the saving of my rent. We are apt to grumble and say it is expensive, but if we only set figures down we should see that at the end of the year we are the gainers. As for the paltry sum of five shillings subscription I should hardly think that those better favoured nurses who have homes would begrudge it, knowing that thus they help their less lucky sisters. As regards the committee it is seldom I send my voting paper back; we do not know the members, and many nurses do the same as I do, therefore it is our own fault, but I do hope that discontent will not enter into our ranks; it only gives other people cause to speak evil of us. We must have rules and I, for one, prefer them too strict rather than too lax.

### A MIDWIFERY CASE.

"Miss E. A. Bedingfeld, a certificated nurse, of Queen Charlotte's Lying-in Hospital," writes: I think the case described by "A. E. H." was one of "false waters." Dr. Ramsbotham, in his *Obstetrical Medicine and Surgery*, says: "Sometimes, though rarely, some fluid is found between the amnion and chorion at an advanced period of gestation, and this is what in labour is called *false waters.*" I have met with several cases of this kind, and at the present time I have a patient who always (I have attended her several times) has a gush of water, with pain and slightly dilated os, a fortnight before real labour sets in. The waters go on escaping for a few days, and then the os closes again. In other cases the rupture has taken place from three to ten days before labour. The pains are usually less regular and pronounced than in real labour, the os being more or less dilated in some cases, in others only patulous. When real labour sets in and the os is well dilated you can often feel distinctly the rent in the chorion through which the "false waters" have escaped. I have noticed that in these cases the amnion is usually very tough and needs to be ruptured artificially. The patient is generally abnormally stout, looking, when six months pregnant, as if she was at her full time. There is often distress in breathing and inability to get about during the last month, owing to the extreme distension, and the relief when the chorion ruptures and the "false waters" escape is very great.

### THE NURSING VOCATION.

"Queen's Nurse" writes: Like your correspondent who writes in your issue for March 30th on "Nursing as a Vocation," I too have watched with much interest the discussion going on for some time past in your pages about nurses and their rights—their proper treatment by patients and by patients' friends. I cannot lay claim, as does your contributor, to thirty years spent in nursing, but I should like to say that my advice, after the much smaller experience of eight years, would be the same as hers—never believe in the words "can't" or "menial." It was a pleasure to read her quiet and dignified article. There are a certain class of nurses who do a sad amount of harm to our noble profession. Nurses and doctors and patients (to their cost!) will recognise the class I refer to when I say that their watchword is "No spoiling of patients or of patients' friends," and to this is added the friendly (?) advice, "Don't work too hard or do anything at all out of the given line. Stand on your dignity and let your patients and their friends see that, though only a nurse, you know what is due to you. Remember your successor: she may not wish to put up with the indignities (?) and worries(?) with which you are now bearing," etc., Now, any nurse who loves her work will at once deny that patients can be spoiled in the sense of being too carefully tended, always provided the tending be judicious—but with regard to the spoiling of patients' friends it is otherwise. Nurses' opinions—as to what is and what is not spoiling them vary very much indeed. Some nurses seem to utterly forget that when sickness invades a house the whole household arrangements become more or less upset, and in their laudable determination not to spoil their patients' friends and to remember their own dignity, they run to extremes. Far different is the behaviour of the nurse who determines to do anything and everything that comes in her way. She will, in my opinion, find that seldom indeed will anything undignified or anything really calculated to injure the status of nurses be asked of her. She brings relief to her patient, gains the confidence of the doctor, and is an untold comfort to her patient's friends. We nurses would do well wherever we find ourselves at the outset to quietly take into consideration all the details of our surroundings and adapt ourselves to them. We may often have occasion "to be very sorry for ourselves"; but if we look our troubles well in the face, and forget there is such a word as grumbling, we shall very soon find ourselves considered as invaluable members of society.

### QUININE PILLS.

"A Radnorshire Nurse" writes: "A case similar to that which was referred to in The Hospital for March 30th occurred when I was engaged in nursing a private patient in Clifton. The patient, a young married lady aged about 23, had suffered from what appeared to be influenza, with a four days' temperature of 103°–104°. On the evening of the fourth day I was called in. One 5gr. quinine pill had been ordered for each evening, and the last had been taken at nine o'clock, just before my arrival. Hot fomentations had also been ordered if the abdominal pains from which the patient had been suffering continued, which they did. After two diarrhœa motions and much pain of a colic nature, most unexpectedly, while putting on a second hot fomentation, the lady was prematurely confined. Everything went on well with the patient; from that evening the temperature was lowered. Then comes the surprising part of the story. After all the natural discharges and motions, after all the fluids which the patient had taken while being treated for influenza, on the fifth or seventh day (I am not certain of the exact number of days, not having the notes at hand just now) six pills, hard and—as described by Dr. Curnow in the article referred to—"apparently unacted on by gastric or intestinal juice," were passed in a fairly loose motion. These pills were coated. At the request of the doctor they were washed free, and dried off on blotting paper and taken (to his great astonishment) to show to the chemist who had dispensed them. He explained that they had been coated in quite the ordinary way. Five pills were out of one box which had contained six, and the last pill taken was one out of a fresh box which had been procured later but made up from the same prescription. Both chemist and doctor appeared to think the fault lay with the pill coating; but as the pills mentioned by Dr. Curnow were not coated, it must have been from some other cause that they failed to dissolve. Evidently, from the surprise shown by both doctor and dispenser, the fact that pills could so pass through the body so completely unchanged had not before come under their notice.

### For Reading to the Sick.

"AS THY DAY THY STRENGTH SHALL BE."

To feel, declining day by day,
Each harsher murmur die away,
And secret springs of joy arise,
To lighten up the weary eyes;
A Hand invisible to feel,
Wounding with kind design to heal;
In every bitter draught to think
Of Him, who learned that cup to drink;
Again and oft again to look
In rapture on that blessèd Book,
Whose soothing words proclaim to thee
That "as thy day thy strength shall be";
Then with changed heart and steadfast mind,
High Heaven before and earth behind,
Thy path of pain again to tread
Till earth receives thy wearied head—
O blessèd lot! who would not raise,
In life or death, the song of *Praise*?—*E. Taylor*.

God takes a thousand times more pains with us than the artist with his picture, by many touches of sorrow, and by many colours of circumstance, to bring man into the form which is the highest and noblest in His sight, if only we received His gifts and myrrh in the right spirit. . . . But when the cup is put away, and these feelings are stifled or unheeded, a greater injury is done to the soul than can ever be amended. For no heart can conceive in what surpassing love God giveth us this myrrh; yet this which we ought to receive to our soul's good, we suffer to pass by us in our sleepy indifference, and nothing comes of it. Then we come and complain: "Alas, Lord! I am so dry, and it is so dark within me!" I tell thee, dear child, open thy heart to the pain, and it will do thee more good than if thou wert full of feeling and devoutness.—*J. Tauler*.

If you have any trial which seems intolerable, pray—pray that it be relieved or changed. There is no harm in that. We may pray for anything, not wrong in itself, with perfect freedom, if we do not pray selfishly. One disabled from duty by sickness may pray for health, that he may do his work; or one hemmed in by internal impediments may pray for utterance, that he may serve better the truth and the right. Or, if we have a besetting sin, we may pray to be delivered from it, in order to serve God and man, and not be ourselves Satans to mislead and destroy. But the answer to the prayer may be, as it was to Paul, not the removal of the thorn, but, instead, a growing insight into its meaning and value. The voice of God in our soul may show us, as we look up to Him, that His strength is enough to enable us to bear it.—*J. F. Clarke*.

O for the peace of a perfect trust,
My loving God, in Thee;
Unwavering faith that never doubts
Thou choosest best for me!

Best, though my plans be all upset;
Best, though my way be rough;
Best, though my earthly store be scant:
In Thee I have enough.

Best, though my health and strength be gone,
Though weary days be mine,
Shut out from much that others have;
Not my will, Lord, but Thine. *E. E. R.*

### Notes and Queries.

**The Editor is always willing to answer in this column, without any fee, all reasonable questions, as soon as possible.**
**But the following rules must be carefully observed:—**
**1. Every communication must be accompanied by the name and address of the writer.**
**2. The question must always bear upon nursing, directly or indirectly.**
**If an answer is required by letter a fee of half-a-crown must be enclosed with the note containing the inquiry.**

*Home for Dying.*

(11) Will you kindly give me the name of a home for the dying, preferably in or near Hampstead, where a woman suffering from heart disease could be taken; she could pay 5s. a week?—*E. F. C.*

The Friedenheim Home of Peace for the Dying, Sunnyside, Upper Avenue Road, Swiss Cottage, N.W.

*Alexandra Nurse.*

(12) Would you tell me where to apply in order to become an Alexandra nurse?—*H. B.*

Apply the Secretary, Soldiers' and Sailors' Families Association, 23 Queen Anne's Gate, Westminster, S.W.

*Domestic Arrangements.*

(13) 1. I should be glad to know if it is usual for district nurses in a nursing home to have jam and marmalade for breakfast and supper in addition to a liberally provided table? 2. Is the servant supposed to brush the nurses' cloaks, &c.—*Marmalade.*

1. Yes, preserves are usually provided. 2. If the nurses require extra personal attention it is usual to make a small charge for it.

*Naval Nursing.*

(14) Sister Edith R. will be glad to know to whom to apply for information respecting Naval Nursing.

The Director General, Medical Department of the Navy, Admiralty, Craven House, Northumberland Avenue, W.C.

*Notice.*

(15) To how long notice is a matron entitled? A nurse generally receives one month; is the time longer in the case of a matron?—*Fever Matron.*

It depends entirely upon the terms of the matron's engagement.

*Private Patient.*

(16) What is the best means of securing a private patient, and what papers in addition to THE HOSPITAL are good for advertising in?—*Nurse K and Nurse.*

If you cannot get patients through private recommendations, your only alternative is to advertise. The usual advertising agents will recommend the most likely papers.

*Nauheim Treatment.*

(17) Is instruction in the Nauheim treatment given in Plymouth, or in any of the provincial towns?—*Nurse H.*

As the Nauheim treatment is given in many private institutions, it is impossible to obtain a comprehensive list of reliable teachers. Apply at your ocal hospital, and see advertisements in medical publications.

*Ergot.*

(18) Will you kindly tell me if a chemist has any right to refuse to sell me Liq. Ext. Ergot? I am a fully-trained and certificated midwife, and have always used it as I was taught. Clause 9 in the L.O.S. Regulations reads: "A midwife may administer or order only such ordinary remedies or drugs as may be required during or after a normal labour."—*A. K. H.*

Ergot of rye and all its preparations are poisons under the Pharmacy Act, and are not to be sold unless the purchaser is known to or is introduced by some person known to the seller, who must at the time of sale enter certain particulars in his poison book. In regard to the question whether the L.O.S. Regulations contemplate the use of ergot by the midwife, A. K. H. had better apply to the Secretary of the London Obstetrical Society, whose certificate we presume she possesses.

*Douches.*

(19) 1. Kindly tell me how to prepare bed and to place patient for uterine examination? 2. Is Higginson's enema syringe, with vaginal nozzle, as effectual as one given with a douche-can? 3. In what position is it best to give douche to an extremely sensitive patient?—*Non-obstetrical.*

For the first question we must refer to the ordinary nursing manuals. 2. Higginson's syringe is even more effectual, if properly used, than the douche-can. The chief advantages of the latter are that it is a safe and convenient arrangement for the patient to use, and that when used by the nurse it leaves one hand at liberty, so the pan, &c., can be more conveniently held in position. 3. The position of course depends to some extent upon what one wants to do. On the whole it is best to have the patient on her back. It must be remembered that when in this position, unless the shoulders be somewhat raised, a certain portion of fluid will be left in the vagina, and will run out as soon as she turns over, unless the nurse is careful to press on the abdomen before removing the pan.

### Standard Books of Reference.

**"The Nursing Profession: How and Where to Train." 2s. net; post free 2s. 4d.**
**"The Nurses' Dictionary of Medical Terms." 2s.**
**"Burdett's Series of Nursing Text-Books." 1s. each.**
**"A Handbook for Nurses." (Illustrated.) 5s.**
**"Nursing: Its Theory and Practice." New Edition. 3s. 6d.**
**"Helps in Sickness and to Health." Fifteenth Thousand. 5s.**
**"The Physiological Feeding of Infants." 1s.**
**"The Physiological Nursery Chart." 1s.; post free, 1s. 3d.**
**"Hospital Expenditure: The Commissariat." 2s. 6d.**
**All these are published by THE SCIENTIFIC PRESS, Ltd., and may be obtained through any bookseller or direct from the publishers, 28 and 29 Southampton Street, London, W.C.**

